# Acute pain and self-directed discharge among hospitalized patients with opioid-related diagnoses: a cohort study

**DOI:** 10.1186/s12954-021-00581-6

**Published:** 2021-12-16

**Authors:** Peggy Compton, Shoshana V. Aronowitz, Heather Klusaritz, Evan Anderson

**Affiliations:** 1 School of Nursing, University of Pennsylvania, Claire Fagin Hall, Room 402, 418 Curie Blvd, Philadelphia, PA 19104 USA; 2 Perelman School of Medicine, University of Pennsylvania, Philadelphia, USA

**Keywords:** Opioids, Pain, Self-directed discharge, Substance use disorders

## Abstract

**Background:**

Patients with substance use disorders are more likely than those without to have a self-directed hospital discharge, putting them at risk for poor health outcomes including progressing illness, readmissions, and death. Inadequate pain management has been identified as a potential motivator of self-directed discharge in this patient population. The objective of this study was to describe the association between acute pain and self-directed discharges among persons with opioid-related conditions; the presence of chronic pain in self-directed discharges was likewise considered.

**Methods:**

We employed a large database of all hospitalizations at acute care hospitals during 2017 in the city of Philadelphia to identify adults with opioid-related conditions and compare the characteristics of admissions ending with routine discharge versus those ending in self-directed discharge. We examined all adult discharges with an ICD-10 diagnoses related to opioid use or poisoning and inspected the diagnostic data to systematically identify acute pain for the listed primary diagnosis and explore patterning in chronic pain diagnoses with respect to discharge outcomes.

**Results:**

Sixteen percent of the 7972 admissions involving opioid-related conditions culminated in self-directed discharge, which was more than five times higher than in the general population. Self-directed discharge rates were positively associated with polysubstance use, nicotine dependence, depression, and homelessness. Among the 955 patients with at least one self-directed discharge, 15.4% had up to 16 additional self-directed discharges during the 12-month observation period. Those admitted with an acutely painful diagnosis were almost twice as likely to complete a self-directed discharge, and for patients with multiple admissions, rates of acutely painful diagnoses increased with each admission coinciding with a cascading pattern of worsening infectious morbidity over time. Chronic pain diagnoses were inconsistent for those patients with multiple admissions, appearing, for the same patient, in one admission but not others; those with inconsistent documentation of chronic pain were substantially more likely to self-discharge.

**Conclusions:**

These findings underscore the importance of pain care in disrupting a process of self-directed discharge, intensifying harm, and preventable financial cost and suffering. Each admission represents a potential opportunity to provide harm reduction and treatment interventions addressing both substance use and pain.

**Supplementary Information:**

The online version contains supplementary material available at 10.1186/s12954-021-00581-6.

## Background

Patients with opioid-related conditions, including opioid dependence and opioid use disorder (OUD), are commonly hospitalized for the treatment of infections, trauma or other emergent conditions, with admission rates increasing as the opioid overdose crisis continues [1–3]. Unfortunately, patients with substance use disorders (SUD) are much more likely to self-discharge against medical advice (referred to in this paper as self-directed discharge [4]) than patients admitted for similar conditions without SUDs [5, 6], which can lead to poorer health outcomes including worsening of illness, readmissions and death [7–9].

‘Discharge in these patients reported as high as 38% [9]. The explanations for this association remain unclear although stigma [10, 11], restrictive hospital policies, and poor pain management [12, 13] may all play a role. In a sample of 480 patients with SUDs in Vancouver, correlates of self-directed discharge included recency of substance use, Indigenous ethnicity (which likely reflects experiences of racism in healthcare settings [14]), and day of week, whereas in-hospital methadone, social support, and older age were protective against self-directed discharge [5].

To explore the role of pain in self-directed discharges, we employed a large non-confidential database of all hospitalizations during 2017 in the city of Philadelphia to identify adults with opioid-related conditions and compare the characteristics of admissions ending with routine discharge versus those ending in self-directed discharge. We applied multiple schemas to the diagnostic data to systematically identify acute pain for the listed primary diagnosis, and explore patterning in chronic pain diagnoses with respect to admission outcomes. Finally, we described characteristics of a subset of patients with multiple self-directed discharges over the course of the year, demonstrating worsening health status and increasing costs over time. The overall goal of these analyses was to explore the role of pain in self-directed discharge and to identify potential approaches to prevent patients who have been historically marginalized from experiencing avoidable harm and suffering.

## Methods

### Design and setting

To examine the relationship between pain and self-directed discharge, we used a cohort study design in which the population of patients admitted to an acute care setting in the city of Philadelphia with an ICD-10 code diagnosis related to opioid use were followed prospectively over a 12-month period. We also examined the role of pain as a risk factor for subsequent self-directed discharge.

### Participants

In this study, we conducted a secondary descriptive analysis of a large database (the Pennsylvania Health Care Cost Containment Council [PHC4]) of all inpatient admissions from all 17 general acute care hospitals in the city of Philadelphia between January 1, 2017 and December 31, 2017. All admissions were retrieved for adults between the ages of 18 and 64 at general acute hospitals with diagnostic codes related to opioid use (F11.10-F11.99, with the exception of F11.11 and F11.21, which refer to use or dependence in remission) or opioid poisoning (T400X1A-T40692A). The study was deemed Exempt (Category 4) from IRB oversight by the Institutional Review Board of the University of Pennsylvania (Protocol # 832523).

The PHC4 is a state agency whose mission is to reduce avoidable healthcare spending. A data record for each inpatient admission is submitted to the PHC4 on a quarterly basis, excluding Veterans Administration Hospitals. To minimize the data collection burden on reporting facilities, the majority of data provided is based on the Uniform Billing standards, thus encounter data are generally extracted from facility billing systems, with multiple bills for the same encounter rolled into a single encounter record. Data are submitted via a secure web-based data upload process. Facilities with overall error rates at or below 15% are provided reports listing data identified as invalid, and asked to make corrections, and facilities with over 15% of their records in error are asked to correct and resubmit their data. Following corrections, the database is closed to any further changes and made available for purchase. With respect to the availability of data and materials, the PHC4 dataset is not publicly available because the data are proprietary in nature.

### Measures

Patient discharge status was determined by a code used to indicate the patient disposition upon discharge as supplied by the facility; those coded as “left against medical advice or discontinued care” were classified as self-directed discharge for the purposes of these analyses. To characterize self-directed discharges, we utilized the ICD-10 diagnosis code indicating the reason for admission as supplied by the facility. The PHC4 data only specifies the quarter of admission, not the exact date, thus to understand the temporal relationship between admissions for patients with multiple discharges, we created an indicator reflecting the standardized-quarter of discharge, with the discharges appearing first indicated by 1 even if occurring in the second or subsequent quarters. For example, the indicators would equal 1, 2, 2, and 3, respectively, for a patient with one discharge in the second quarter, two in the third quarter and one in the fourth quarter.

Demographics of the sample considered in the analysis include race, ethnicity, gender, and age. Housing instability was identified by diagnostic codes for homelessness, housing insecurity, and problems related to housing and economic circumstances. Patients were coded as nicotine dependent if they had an ICD indicator for nicotine dependence during at least one hospital admission and were coded as polysubstance users if they had dependence or poisoning for cannabis, cocaine, stimulants, or depressants noted during at least one hospital admission. To explore the role of infectious pathologies we separated a list of injection-related infections developed by Coye and colleagues into three subcategories: skin and soft tissue infections, sepsis and bacteremia, or bone, joint and vascular infections [15] (see Table 1 and Additional file 1 for additional details about associated ICD-10 codes).

**Table 1:** Characteristics of admissions by discharge type (routine versus self-directed) in Philadelphia general acute hospitals for patients with diagnosed opioid dependence or poisoning, 2017

	Total	Routine	Self-directed	*p*
All admissions (N)	7972	6728	1244	
Median length of stay (IQR)	4 (2–6)	4 (2–7)	2 (1–3)	< .0001
Male	4426 (55.5)	3653 (54.3)	773 (62.1)	< .0001
Average age (Std)	41.44 (12.1)	41.89 (12.3)	38.97 (11.0)	< .0001
Homeless^a^	615 (7.7)	479 (7.1)	136 (10.9)	< .0001
Acute pain^e^	2856 (35.8)	2275 (33.8)	581 (46.7)	< .0001
Major chronic pain^b^	768 (9.6)	694 (10.3)	74 (5.9)	< .0001
Mikosz chronic pain^c^	915 (11.5)	807 (12.0)	108 (8.7)	< .0006
Chronic overlapping pain conditions^d^	467 (5.9)	408 (6.1)	59 (4.7)	0.0757
*Injection related infection*^f^				
Skin and soft tissue infection	1248 (15.7)	886 (13.2)	362 (29.1)	< .0001
Septicemia or bacteremia infection	947 (11.9)	751 (11.2)	196 (15.8)	< .0001
Bone, joint, or vascular infection	470 (5.9)	349 (5.2)	121 (9.7)	< .0001
Polysubstance use^g^	3422 (42.9)	2760 (41.0)	662 (53.2)	< .0001
Nicotine dependent^h^	5106 (64.1)	4206 (62.5)	900 (72.4)	< .0001
Patients with 2+ admissions (N)	3287	2616	671	
Major chronic pain^b^	309 (9.4)	262 (10.0)	47 (7.0)	0.0171
“Missing” major chronic pain^b^	367 (11.2)	278 (10.6)	89 (13.3)	0.0530
Mikosz chronic pain^c^	352 (10.7)	287 (11.0)	65 (9.7)	0.3373
“Missing” Mikosz chronic pain^c^	392 (11.9)	280 (10.7)	112 (16.7)	0.0001
Chronic Overlapping Pain Conditions^d^	173 (5.3)	138 (5.3)	35 (5.2)	0.9512
“Missing” COPC	251 (7.6)	180 (6.9)	71 (10.6)	0.0013

Data are expressed as No. (%) unless otherwise indicated

^a^Indicates homelessness, housing insecurity, and problems related to housing and economic circumstances. The full list of associated ICD-10 codes is provided in Additional file 1

^b^Indicates central pain syndrome, chronic pain, neoplasm-related pain, and chronic pain syndrome

^c^Indicates chronic nonradicular and radicular back pain, chronic neck pain, fibromyalgia, inflammatory joint disorders, irritable bowel syndrome, non-migraine headaches, osteoarthritis and joint cartilage conditions, and periarticular/soft tissue disorders

^d^Indicates fibromyalgia, irritable bowel syndrome, interstitial cystitis/bladder pain syndrome, chronic prostatitis, vulvodynia, migraine, chronic tension-type headache, temporomandibular disorder, chronic low back pain, chronic fatigue syndrome, endometriosis with pain

^e^Indicates acute pain based on independent coding of admitting diagnoses by two registered nurses

^f^Indicates injection related infections separated into skin and soft tissue infections (abscess, cellulitis, necrotizing fasciitis), septicemia and bacteremia, and bone, joint and vascular infections (septic pulmonary embolism, endocarditis, staphylococcal arthritis, osteomyelitis, and intraspinal abscess.)

^g^Indicates use of or poisoning from cannabis, cocaine, stimulates, sedatives, hypnotic or anxiolytics, hallucinogens,

^h^Indicates current nicotine dependence and a history of nicotine dependence

To identify acute pain in the cohort, two PhD-prepared registered nurses with extensive clinical expertise in substance use and acute pain independently coded each primary diagnosis as being either acutely painful or not acutely painful. Initial agreement between the nurse coders was 91%; the remaining discrepant cases were resolved upon discussion. The majority of acutely painful conditions were traumatic, infectious, or inflammatory in origin. To explore the role of chronic pain in self-directed discharges, we created an indicator measuring the presence of any of the major chronic pain diagnoses: central pain syndrome, chronic pain, neoplasm-related pain, and chronic pain syndrome. Given evidence that these fail to capture many types of chronic pain, we also created an indicator for chronic pain based on a schema deployed by Mikosz and colleagues [16], which includes ICD-10 codes for chronic non-radicular and radicular back pain, chronic neck pain, fibromyalgia, inflammatory joint disorders, irritable bowel syndrome, non-migraine headaches, osteoarthritis and joint cartilage conditions, and periarticular/soft tissue disorders. We additionally used the schema from Schrepf and colleagues to identify Chronic Overlapping Pain Conditions (COPC) [17].

### Data analysis

To examine differences in admissions based on whether discharge was routine or self-directed, we conducted Fisher exact tests on dichotomous indicators for sex, housing instability, and acute pain, and a t-test and a Wilcoxon rank sum test on continuous measures of age and length of stay (LOS). We explored patterning in self-directed discharge rates based on the presence of acute and chronic pain indictors with Chi-square tests. Logistic regression was conducted to understand how chronic and acute pain indicators correlate with self-directed discharge, adjusting for LOS, sex, age, and housing insecurity. Finally, because a substantial number of patients experienced more than one admission during the 12-month observation period, we described as groups those patients with zero, one, two to five, and six or more self-directed discharges. Cochran-Armitage Trend Tests were conducted to explore the dose response relationship between number of self-directed discharges and patient characteristics. Three case studies are provided to exemplify the progression of pain and disease across multiple self-directed discharges. Inconsistent documentation of chronic pain in those patients with multiple diagnoses was noted and the degree to which these “missing” diagnoses predicted self-directed discharge also inspected. Analyses were completed using SAS 9.4 (SAS Institute, Cary NC).

## Results

**Fig. 1:**
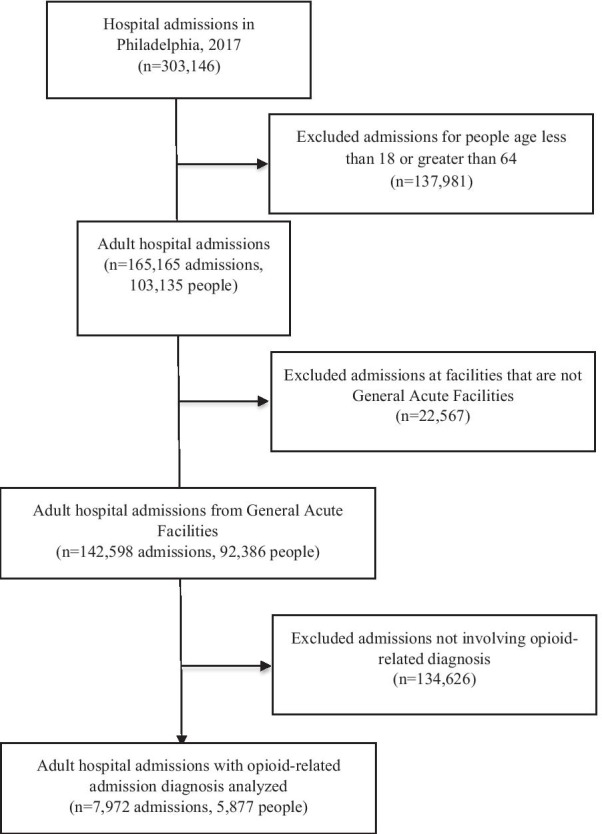
CONSORT flow diagram

Of the 165,165 admissions to general acute care hospitals during the observation period among people ages 18–64, 7972 (4.8%) involved an opioid-related condition among 5877 unique patients (see Fig. 1). Rates of self-directed discharges were 5.5 times higher in those with an opioid-related condition compared to those without (15.6% vs. 2.8%). Self-directed discharges were more likely among individuals identified as male-gender than those identified as female gender (17.5% vs 13.3%; *p* < 0.0001), and those with self-directed discharge were also significantly younger (39.0 yrs; SD, 11.0 vs. 41.9 yrs; SD, 12.3; *p* < 0.0001). As anticipated, median LOS was significantly shorter for those with self-directed discharge in comparison to routine discharge (2 vs. 4 days; IQR 1–3 vs. 2–7; *p* < 0.0001) (Table 1).

**Fig. 2:**
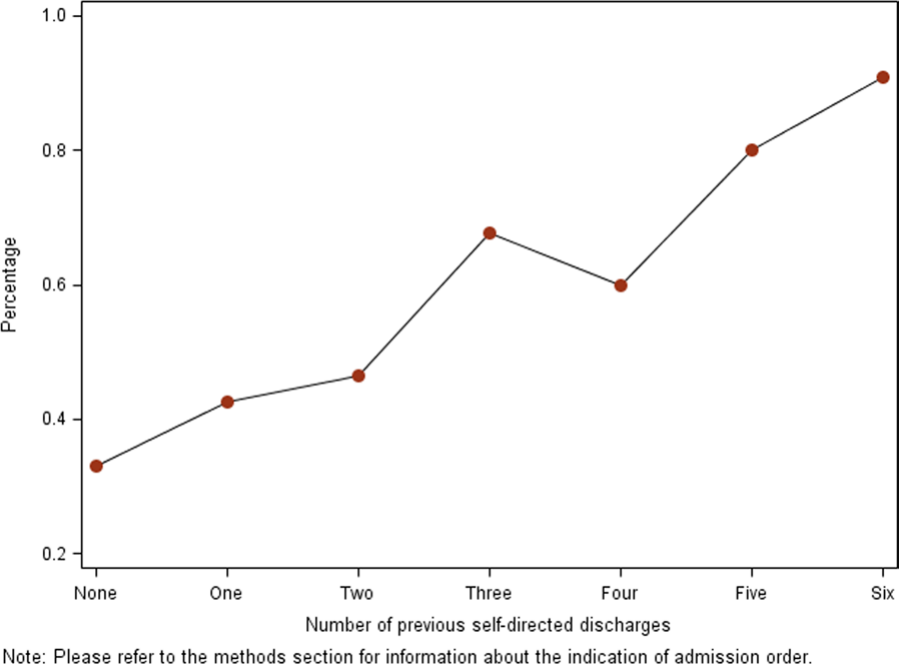
Percentage of admissions involving acute pain by number of previous self-directed discharges

Just over a third (36%) of all primary admitting diagnoses were identified as acutely painful. Patients with acute pain were over 1.5 times as likely to have a self-directed discharge (20.3%) as patients without an acutely painful primary diagnosis (13.0%). For those patients with multiple admissions, the presence of acute pain increased dramatically and consistently as patients accumulated self-directed discharges; for patients with six previous self-directed discharges, 90% of the admitting diagnosis were acutely painful (Fig. 2).

Chronic pain was documented in 9.6% and 11.5% of all admissions depending on the measure used, and COPC were present in 5.9% of all admissions. For patients with multiple self-directed discharges, diagnoses across all three chronic pain measures were inconsistent in that, for the same patient, it was documented for some admissions but not all previous or subsequent admissions. Self-directed discharge was between 1.4 and 1.6 times more common for multiple admissions with inconsistently documented chronic pain indicators compared with multiple admissions with consistently documented chronic pain (15.2% vs 24.3%; 18.5% vs. 28.6%; 20.2% vs. 28.3%). Among those with multiple admissions, missing chronic pain indicators were substantially more common among patients with unstable housing (25.1% vs 19.1% for major chromic pain; 31.4% vs 20.7% for Mikosz chronic pain; 32.7% vs 19.1% for overlapping chronic pain, all *p* < 0.0001) (Table 2).

**Table 2:** Characteristics of admissions by presence of acute and chronic pain measures

	Admissions *N* (%)	Homeless *N* (%)	LOS days^a^ Median (IQR)	Self-discharge *N* (%)
All admissions	7972	985 (12.4%)	4 (2–6)	1244 (15.6%)
Acute pain	2856 (35.8%)	471 (16.5%)	4 (2–8)	581 (20.3%)
No acute pain	5116 (64.2%)	514 (10.1%)	4 (2–6)	663 (12.9%)
Admissions for patients with 2+ admissions	3287	701 (21.3%)	4 (2–7)	671 (20.4%)
*Major chronic pain*^a^				
Neither documented nor missing	2611 (79.4%)	550 (21.1%)	4 (2–6)	535 (20.5%)
Documented	309 (9.4%)	59 (19.1%)	4 (2–7)	47 (15.2%)
“Missing”	367 (11.2%)	92 (25.1%)	4 (2–9)	89 (24.3%)
*Mikosz chronic pain*^a^				
Neither documented nor missing	2543 (77.4%)	505 (19.9%)	4 (2–6)	494 (19.4%)
Documented	352 (10.7%)	73 (20.7%)	4 (2–8)	65 (18.5%)
“Missing”	392 (11.9%)	123 (31.4%)	4 (2–7)	112 (28.6%)
*Chronic overlapping pain conditions*^a^				
Neither documented nor missing	2863 (87.1%)	586 (20.5%)	4 (2–6)	565 (19.7%)
Documented	173 (5.3%)	33 (19.1%)	4 (2–7)	35 (20.2%)
“Missing”	251 (7.6%)	82 (32.7%)	4 (2–7)	71 (28.3%)

The full list of associated ICD-10 codes is provided in Additional file 1. Data are expressed as No. (%) unless otherwise indicated. Additional information about indicators is provide in Table

^a^Length of stay expressed as average (Std)

**Fig. 3:**
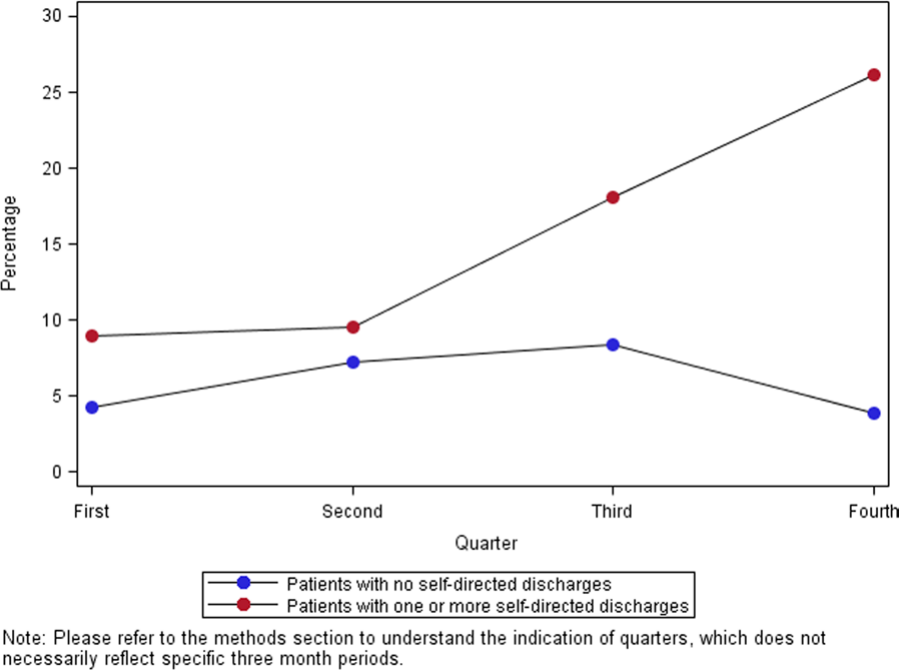
Percentage of admissions involving bone, joint, or vascular injection-related infection by order and patient self-directed discharge history

Among the 955 patients with at least one self-directed discharge, 147 (15.4%) had between one and 16 additional self-directed discharges. There was a positive and graded relationship of the number of self-directed discharges to infections, polysubstance use, homelessness, nicotine dependence, depression and average total spending (Table 3). Patients with a history of multiple self-directed discharges experienced dramatically more injection-related infections in their bones, joints and vascular system as the year advanced (Fig. 3). Given that the underlying data only identifies the quarter of admission, it was only possible to definitively identify 392 admissions occurring after a self-directed discharge within the 1-year observation period. Bone, joint, and vascular system infections were three times more likely in these 392 admissions (*n* = 58; 14.8%) compared to the 7138 admissions definitely not following a self-directed discharge (*n* = 339; 4.7%). Hospitalizations involving bone, joint, and vascular infections were almost twice as costly on average as hospitalizations involving skin-related infections ($183,690 vs $97,608; *p* < 0.0001).

**Table 3:** Patient characteristics by number of self-directed discharges in 2017

	Number of times self-discharging in 2017				Cochran-armitage trend test
	None	1	2–5	6+	
N (%)	4922 (83.75%)	808 (13.75%)	137 (2.33%)	10 (0.17%)	
White	2720 (55.31%)	504 (62.45%)	95 (69.34%)	9 (90%)	
Black	1483 (30.15%)	176 (21.81%)	20 (14.60%)	1 (10%)	
Race other^a^	392 (7.97%)	82 (10.16%)	11 (8.03%)	0	
Race unknown	324 (6.59%)	45 (5.58%)	11 (8.03%)	0	
Patient is of Hispanic/Latin descent	370 (7.52)	72 (8.91)	8 (5.84)	0	
Male	2691 (54.68%)	505 (62.50%)	89 (64.96%)	6 (60.00%)	
Age (average, Std)	42.00 (12.35)	38.53 (11.08)	39.71 (10.44)	40.70 (12.05)	
Ever^b^ homeless^c^	361 (7.34%)	93 (11.51%)	37 (27.01%)	6 (60%)	< .0001
Ever skin and soft tissue infection	660 (13.41%)	226 (27.97%)	68 (49.64%)	9 (90%)	< .0001
Ever septicemia or bacteremia infection	612 (12.44%)	143 (17.70%)	55 (40.15%)	7 (70%)	< .0001
Ever bone, joint, or vascular infection	234 (4.76%)	63 (7.80%)	32 (23.36%)	7 (70%)	< .0001
Ever any polysubstance use	2139 (43.47%)	493 (61.01%)	101 (73.72%)	10 (100%)	< .0001
Ever depression	2188 (44.45)	328 (40.59)	83 (60.58)	10 (100)	< .0001
Ever cocaine use	1347 (27.37%)	339 (41.96%)	85 (62.04%)	10 (100%)	< .0001
Ever nicotine dependent	3151 (64.04%)	604 (74.75%)	131 (95.62%)	10 (100%)	< .0001
Charges for all 2017 admissions (average, Std)	$ 103,625 ($162,150)	$ 83,809 ($149,493)	$ 245,596 ($304,555)	$ 426,912 ($276,882)	

Data are expressed as No. (%) unless otherwise indicated

^a^The race indicator is also missing for four people

^b^Ever measures refer to the presence of the indicator during at least one patient admission in 2017

^c^Additional information about indicators is provide in Table 1. The full list of associated ICD-10 codes is provided in Additional file 1

The 392 hospitalizations which definitely occurred after a self-directed discharge, resulted in charges totaling $26,040,983, for which the primary payer was overwhelmingly Medicaid (79.4%) or Medicare (9.2%). Another 442 discharges occurred in the same quarter as—and therefore potentially after—a self-directed discharge, and resulted in hospitalization charges totaling $26,081,336, with Medicaid (75.6%) and Medicare (13.8%) once again the primary source of reimbursement. Patients were identified as lacking health insurance in only 0.5% of these 834 discharges.

**Fig. 4:**
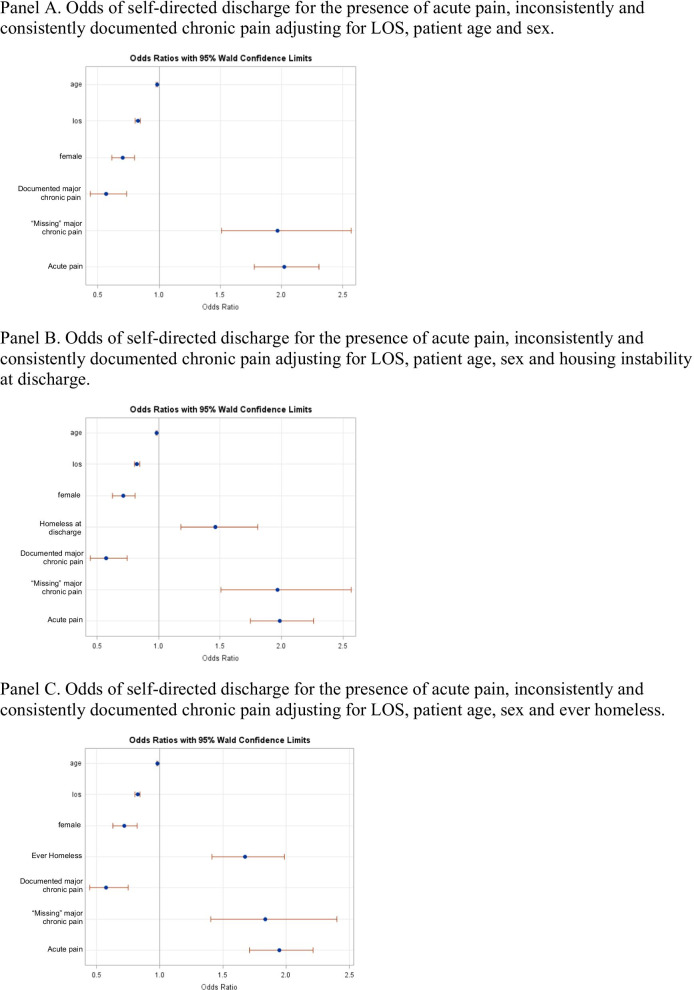
Logistic regression models for odds of self-directed discharge. **A** Odds of self-directed discharge for the presence of acute pain, inconsistently and consistently documented chronic pain adjusting for LOS, patient age and sex. **B** Odds of self-directed discharge for the presence of acute pain, inconsistently and consistently documented chronic pain adjusting for LOS, patient age, sex and housing instability at discharge. **C** Odds of self-directed discharge for the presence of acute pain, inconsistently and consistently documented chronic pain adjusting for LOS, patient age, sex and ever homeless

A major chronic pain diagnosis reduced the odds of self-directed discharge (OR = 0.57, 95%CI 0.44–0.73), while the presence of acute pain (OR = 2.03, 95%CI 1.78–2.30, Table 3, Fig. 4A) and inconsistently documented chronic pain (OR = 1.97, 95%CI 1.51–2.57) did the opposite, after adjusting for LOS, patient age and sex. Adding a covariate measuring whether housing instability was identified at the specific admission did not substantially alter the estimates and confidence levels (Table 3, Fig. 4B), however adding a covariate indicating any history of homelessness in the year for the patient partially attenuated the effect of missing chronic and acute pain, yet both retained significance (OR = 1.84, 95%CI 1.40–2.40, OR = 1.95, 95%CI1.71–2.22, Table 3, Fig. 4C).

As noted above, over 15% of patients had more than one self-directed discharge in 2017. To better characterize these individuals, we provide exemplar case studies including admitting diagnoses and other medical and psychiatric conditions, procedures administered, lengths of stay, and estimated associated hospitalization costs. Due to limitations in the database, time intervals between self-directed discharges were not available, but self-directed discharges are ordered by 3-month quarters.

### Case study #1

Patient was admitted to a general acute care hospital 15 times in 2017, twice through urgent care and the remainder of times through the emergency room. Ten of the admissions resulted in a self-directed discharge, with lengths of stay for those discharges ranging from 0 to 6 days. Although the patient had opioid use disorder and opioid dependence diagnoses, none of the admissions were related to overdose. All but one of the admissions was for a painful condition. Admitting diagnoses progressed from osteomyelitis of the hand (×2) to bacteremia (×2) to sepsis due to streptococcus (×2) to sepsis due to methicillin resistant Staphylococcus aureus. Intervening admissions were related to cellulitis of the right (×1) and left (×2) lower limbs and acute embolism and thrombosis of right (×1), left (×1) and bilateral (×1) femoral veins. Procedures administered included transesophageal ultrasound of the heart and aorta, superior vena cava infusion, and drainage of the left knee joint. The patient was identified as having nicotine and cocaine dependence in addition to opioid use disorder and opioid dependence diagnoses. The patient also had diagnosis of chronic hepatitis C, major depressive disorder, and was noted to be experiencing homelessness on 5 admissions. One admission related to a “fall down embankment” and another related to an assault. Payment for this patient’s hospitalization was provided by Medicaid, Blue Cros,s, and government sources and total costs associated with the care received was $775,823.46.

### Case study #2

Patient was admitted to a general acute care hospital 20 times during the first 9 months of 2017, three times through urgent care and the remainder through the emergency room. Seventeen of the admissions resulted in a self-directed discharge, with lengths of stay for those discharges ranging from 0 to 13 days. The patient had an opioid dependence diagnosis, and one admission was related to overdose; the patient was also diagnosed with cocaine use disorder and nicotine dependence. All but one admission was for a painful condition. Admitting diagnoses progressed from cellulitis of the left (×8) and right (×2) upper limb, to extradural and subdural abscess (×2), to intraspinal abscess and granuloma (×1), to sepsis due to methicillin resistant Staphylococcus aureus (×2). The patient’s course was complicated by insulin-dependent Type 2 diabetes with hyperglycemia, and two admissions were related to open wounds and chronic skin ulcers. Procedures administered included superior vena cava infusion and drainage of the intracranial epidural space and of the buttock. On several admissions, anxiety disorder and major depressive disorder were identified. One admission also related directly to a fall. This patient had Medicaid insurance coverage and total costs associated with hospitalization was $871,666.50.

### Case study #3

Patient was admitted to a general acute care hospital 16 times in 2017, each time through the emergency room. All but the first discharge was a self-directed discharge, with lengths of stay for those discharges ranging from 0 to 3 days. Although the patient had opioid use disorder and opioid dependence diagnoses, none of the admissions were related to overdose. All his admissions were for painful, infectious conditions. Specifically, the patient was admitted with a primary or secondary diagnosis of cellulitis of the right lower extremity nine times, which progressed to an osteomyelitis of the right tibia by the 12th admission. Procedures administered include bronchial endoscopy and cardiac fluoroscopy. Also noted were diagnoses of cocaine use disorder, cannabis dependence, alcohol dependence, and nicotine dependence; on eight of the admissions, the patient was noted to be experiencing homelessness. This patient had Medicaid insurance coverage and total hospitalization costs associated with care received was $490,742.71.

## Discussion

The results of these analyses extend previous research documenting high rates of self-directed discharges among individuals with SUD [9, 18]. In this cohort of patients admitted to acute care hospitals, patients with opioid-related diagnoses most commonly presented with infectious conditions, although trauma and overdose also accounted for admissions. As illustrated in the case studies, over time infections worsened, progressing from those of skin and soft tissue (i.e., cellulitis, abscess) to more pervasive (i.e., endocarditis, osteomyelitis) or systemic (i.e., sepsis) across hospital discharges. National trends indicate a rise in these infections [19], however they are often inadequately treated as a result of structural barriers [20, 21]. Self-directed discharge is especially harmful for patients with skin and soft tissue infections, which must be fully treated to avoid recurrence or further colonization in bones, joints, and the vascular system. While providing high quality treatment for acute needs, these hospital touchpoints are also an important opportunity for immediate linkages to evidence-based treatment for patients. To reduce costs and worsening morbidity, interventions aimed at reducing the harms associated with injection substance use [22] should be consistently offered to hospitalized patients with OUD, including but not limited to evidence-based treatment with medications like methadone and buprenorphine [23]. This may be especially effective if delivered to patients needing prolonged hospitalization [24].

It is likely that the factors that result in self-directed discharges from the hospital also keep patients from seeking care in these settings, allowing infections to worsen prior to treatment [25]. People who inject drugs report self-treating infections by lancing wounds, taking antibiotics not prescribed to them, and treating pain with street-purchased opioids [26] in order to avoid traditional healthcare settings. A promising intervention is community-based wound care co-located at syringe access programs that provide comprehensive and compassionate wound treatment for patients who inject drugs to help prevent infections from progressing to a point where inpatient care is necessary [27].

Of those with multiple self-directed discharges, polysubstance use and nicotine dependence were common; these patients also had higher rates of homelessness than persons who did not self-discharge. Consistent with the literature, polysubstance (including nicotine) use is extremely common among persons with SUD [28], as are high rates of comorbid depression [29]. In addition, SUD and housing insecurity are often related, with SUD increasing the risk of homelessness in some populations, as well as unstable housing leading to increased substance use [30–32]. Of note, many of the patients completed a self-directed discharge despite not having stable housing, even during quarters with inclement Philadelphia weather. Overall, patients admitted to acute care hospitals with opioid-related diagnoses who completed a self-directed discharged were representative of individuals with un- or under-treated SUD.

Poorly treated pain is often cited as a reason why patients with SUD engage in a self-directed discharge [33–35]. This was reflected in our findings, and aligns with qualitative work suggesting that the pain of hospitalized patients with SUD is not seriously or respectfully addressed [36]. Patients with acutely painful admitting diagnoses were almost twice as likely to have a self-directed discharge than those without, and these diagnosis codes were extremely common among patients who had a self-directed discharge on six or more occasions. In addition, for those with multiple admissions, self-directed discharge was also substantially more common for admissions with inconsistent documentation of chronic pain compared with multiple admissions with documented chronic pain, and unstably housed patients had higher rates of “missing” chronic pain indicators than patients not identified as unstably housed. These findings suggest that untreated or unrecognized (and thus undocumented) chronic pain might play a role in patients’ decisions to engage in a self-directed discharge, and that patients experiencing homelessness might be especially vulnerable to under-recognition of chronic pain conditions. Because we were unable to assess whether inconsistent chronic pain indicators were due to chronic pain conditions in remission or resolved, future research should focus on the assessment of chronic pain conditions among patients with SUD, and especially those experiencing housing unstability. Stigma towards persons with SUDs, including OUD, may account for an under-assessment and documentation of pain [37]. Our findings suggest that a thorough assessment and treatment of acute and chronic pain may decrease rates of self-directed discharge among these marginalized patients [38]. Anti-stigma training may be one important intervention to address reduce stigma towards persons with SUDs among healthcare providers [39, 40].

A substantial minority of the sample (15.4%) had multiple self-directed discharges over the course of the 12-month observation period. Rates of polysubstance use, nicotine dependence, and depression increased with the number of self-directed discharges, and were present in 100% of patients who had six or more self-directed discharges. Addressing the overlapping mental health and social vulnerabilities of these patients is challenging and incompletely addressed within existing acute care models. The imbedding of addiction medicine specialists within the acute care hospital setting may be one approach to address some of these concerns. However, research suggests that the benefits of such programs may be limited [41] unless accompanied by a transformation in the “macro‐ and community‐level systems and institutions that drive social, political, and economic disadvantage and health inequities” [42]. Financing these transformations through new delivery models and other community-based investments makes sense from a tax-payer perspective; we conservatively identified tens of millions of dollars of hospital spending, financed overwhelmingly by Medicaid and Medicare, for admissions occurring after a self-directed discharge. It is unclear whether the same financial logic would incentivize hospitals; less than 1% of patients with repeat self-directed discharges lacked insurance. However, hospital investments in community-based interventions could be explicitly tied to the federally mandated community health needs assessment and implementation plans [43]. Although readmission penalties have complicated and contested effects for patients with cardiovascular disease [44], similar financial inducements may be necessary for hospitals to embrace practices to improve care for this patient population.

Although pain and untreated OUD appear to be related to self-directed discharge in this cohort, it is likely the result of multiple intersecting factors. Significant numbers of persons self-discharging are nicotine dependent, and due to strict non-smoking policies in hospital settings, nicotine withdrawal or craving may motivate self-directed discharge [45]. Similarly, many of these patients use multiple substances, and not captured in these data is the degree to which benzodiazepine, sedative or alcohol withdrawal might explain self-directed discharge. As previously mentioned, many health care providers hold stigmatizing views of persons with SUD [46–48], which may translate into less than empathetic or even discriminatory care [49]. These patients may be subject to especially restrictive hospital policies with respect to visitors or urine testing. Repeated negative or traumatic experiences in the hospital environment likely contribute to departure from the inpatient setting.

### Limitations

The primary limitation of the study is reliance on high-level coded data, precluding nuanced understanding of patient experiences and why they chose self-directed discharge. In addition, the opioid-related diagnostic codes are not specific to OUD, thus patients who did not meet diagnostic criteria for a SUD were likely included in the sample. The data are also limited to 1 year providing only partial visibility into the hospitalization history of the patient population. Although coded by independent nurse experts, there may have been errors in conditions classified as acutely painful. Moreover, only the primary diagnoses were assessed as acutely painful; patients admitted with a non-acutely painful primary diagnosis (i.e., overdose) may have also had co-occurring acute pain from another condition (i.e., abscess). Further complicating interpretation in this opioid-using sample is the potential confounding effect of opioid-induced hyperalgesia [50], such that persons whose conditions were coded as not painful by the investigators may have in fact been experienced as painful by the patient. So-called “missing” chronic pain diagnoses could not be identified in those patients with only a single admission. These sources of misclassification bias suggest that the observed estimates underrepresent the incidence and role of pain in self-directed discharges. Finally, we did not have access to information about analgesic use, thus could not assess if and how painful conditions were treated.

## Conclusion

Individuals admitted to the hospital who have an opioid-related diagnosis are more likely than those without to experience self-directed discharge. Rates of self-directed discharge likely reflect unrecognized or poorly managed pain. Notably, a significant number of these patients experience multiple and repeated self-directed discharges from acute care settings, with worsening morbidity and increased cost over time. Clear from these findings is the phenomenally high cost of inadequate care represented by self-directed discharge and short hospital stays for patients who require longer-term in-patient care, suggesting the need for substantial reform in the delivery of acute care. In that each hospital admission represents a potential opportunity to provide harm reduction and treatment interventions addressing both substance use and pain, self-directed discharges related to un- or under-treated SUD as well as acute and chronic pain would likely decline if these were consistently and compassionately provided.

## Supplementary Information

**Additional file 1**. ICD-10 Codes used to identify opioid poisoning, opioid dependence, chronic pain indicators, chronic overlpping pain conditions, infection, homelessness, other substance use and depression.

## Data Availability

As noted in the ‘Availability of data and materials’ statement in the *Participants* section of the manuscript, the dataset analyzed during the current study are not publicly available because the data are proprietary in nature.
