# Supplementary material for: Acute pain and self-directed discharge among hospitalized patients with opioid-related diagnoses: a cohort study
Source: Harm Reduct J. 2021 Dec 16;18:131. doi: 10.1186/s12954-021-00581-6 (PMC8679978; doi:10.1186/s12954-021-00581-6)
Supplement: Supplementary file 1 — Additional file 1. ICD-10 Codes used to identify opioid poisoning, opioid dependence, chronic pain indicators, chronic overlpping pain conditions, infection, homelessness, other substance use and depression. [file 12954_2021_581_MOESM1_ESM.docx]

Supplement 1.

| Category | ICD-10 codes |
| --- | --- |
| Opioid poisoning | F11, F111, F1110, F1112, F11120, F11121, F11122, F11129, F1114, F1115, F11150, F11151, F11159, F1118, F11181, F11182, F11188, F1119, F112, F1120, F1122, F11220, F11221, F11222, F11229, F1123, F1124, F1125, F11250, F11251, F1129, F11259, F1128, F11281, F11282, F11288, F119, F1192, F11920, F11921, F11922, F11929, F1193, F1194, F1195, F11950, F11951, F11959, F1198, F11981, F11982, F11988, F1199 |
| Opioid dependence | T40, T400, T400X, T400X1, T400X1A, T400X1D, T400X1S, T400X2, T400X2A, T400X2D, T400X2S, T400X3, T400X3A, T400X3D, T400X3S, T400X4, T400X4A, T400X4D, T400X4S, T400X5, T400X5A, T400X5D, T400X5S, T401, T401D, T401X, T401X1, T401X1A, T401X1D, T401X1S, T401X2, T401X2A, T401X2D, T401X2S, T401X3, T401X3A, T401X3D, T401X3S, T401X4, T401X4A, T401X4D, T401X4S, T402, T402X, T402X1, T402X1A, T402X1D, T402X1S, T402X2, T402X2A, T402X2D, T402X2S, T402X3, T402X3A, T402X3D, T402X3S, T402X4, T402X4A, T402X4D, T402X4S, T402X5, T402X5A, T402X5D, T402X5S, T403, T403X, T403X1, T403X1A, T403X1D, T403X1S, T403X2, T403X2A, T403X2D, T403X2S, T403X3, T403X3A, T403X3D, T403X3S, T403X4, T403X4A, T403X4D, T403X5, T403X5A, T403X5D, T403X5S, T404, T404X, T404X1, T404X1A, T404X1S, T404X2, T404X2A, T404X2D, T404X2S, T404X3, T404X3A, T404X3D, T404X3S, T404X4, T404X4A, T404X4D, T404X4S, T404X5A, T404X5D, T404X5S |
| Major Chronic pain | G890, G8921, G8928, G8929, G894 |
| Chronic pain indicators from Mikosz CA, Zhang K, Haegerich T, et al. Indication-Specific Opioid Prescribing for US Patients With Medicaid or Private Insurance, 2017. JAMA Netw Open. 2020;3(5). | M2578, M4000, M4003, M4004, M4005, M40202, M40203, M40204, M40205, M40209, M40292, M40293, M40294, M40295, M40299, M4030, M4035, M4036, M4037, M4100, M4102, M4103, M4104, M4105, M4106, M4107, M4108, M41112, M41113, M41114, M41115, M41116, M41117, M41119, M41122, M41123, M41124, M41125, M41126, M41127, M41129, M4120, M4122, M4123, M4124, M4125, M4126, M4127, M4130, M4134, M4135, M4180, M4182, M4183, M4184, M4185, M4186, M4187, M419, M4300, M4301, M4302, M4303, M4304, M4305, M4306, M4307, M4308, M4309, M4310, M4311, M4312, M4313, M4314, M4315, M4316, M4317, M4318, M4319, M4320, M4321, M4322, M4323, M4324, M4325, M4326, M4327, M4328, M438X9, M4640, M4644, M4645, M4646, M4647, M4648, M4649, M4710, M4714, M4715, M4716, M4720, M47814, M47815, M47816, M47817, M47818, M47819, M47894, M47895, M47896, M47897, M47898, M47899, M479, M4800, M4804, M4805, M4806, M48061, M48062, M4807, M4808, M4810, M4811, M4812, M4813, M4814, M4815, M4816, M4817, M4818, M4819, M4820, M4821, M4822, M4823, M4824, M4825, M4826, M4827, M4830, M4831, M4832, M4833, M4834, M4835, M4836, M4837, M4838, M489, M5104, M5105, M5106, M5124, M5125, M5126, M5127, M5134, M5135, M5136, M5137, M5146, M5147, M5184, M5185, M5186, M5187, M519, M532X7, M532X8, M533, M5380, M5384, M5385, M5386, M5387, M5388, M539, M5403, M5404, M5405, M5406, M5407, M5408, M5409, M5430, M5431, M5432, M545, M546, M5489, M549, M62830, M961, M962, M963, M965, M9922, M9923, M9924, M9925, M9926, M9927, M9928, M9929, M9932, M9933, M9934, M9935, M9936, M9937, M9938, M9939, M9942, M9943, M9944, M9945, M9946, M9947, M9948, M9949, M9952, M9953, M9954, M9955, M9956, M9957, M9958, M9959, M9962, M9963, M9964, M9965, M9966, M9967, M9968, M9969, M9972, M9973, M9974, M9975, M9976, M9977, M9978, M9979, M9983, M9984, M9902, M9904, Q762, M4724, M4725, M4726, M4727, M4728, M5114, M5115, M5116, M5117, M5414, M5415, M5416, M5417, M5418, M5440, M5441, M5442, M47811, M47891, M4723, M4722, M4721, M47813, M47893, M47892, M47812, M47012, M4711, M47014, M47011, M4712, M47022, M47029, M47019, M47016, M47013, M47021, M4713, M47015, M50220, M5022, M5023, M5020, M50221, M50222, M50223, M5021, M5002, M5000, M5001, M50022, M50023, M50021, M50020, M5003, M5082, M5091, M5012, M50922, M50821, M5080, M5011, M50123, M50822, M50820, M50921, M5010, M4643, M5093, M50120, M50823, M5083, M50121, M5090, M4641, M50920, M50122, M4642, M5092, M50923, M5013, M5081, M9930, M9971, M9921, M9970, M4803, M9931, M9940, M9960, M9950, M4802, M9951, M4801, M9961, M9941, M9920, M542, M530, M531, M5413, M5411, M5412, M436, M5400, M5402, M5401, M5382, M5481, M5381, M5383, M797, M05, M06, M08, M120, M255, M353, M45, M460, M461, M465, M468, M469, M488X, M498, M790, K58, G44009, G44001, G44019, G44011, G44021, G44029, G44031, G44039, G44041, G44049, G44059, G44051, G44091, G44099, G44201, G44211, G44219, G44229, G44221, R51, G441, M129, M131, M138, M15, M16, M17, M18, M19, M221, M222, M223, M224, M228, M229, M23, M241, M7500, M7501, M7502, M2570, M2571, M2572, M2573, M2574, M2575, M2576, M2577, M66211, M66212, M66219, M66811, M66812, M66819, M701, M702, M704, M705, M706, M707, M751, M752, M753, M754, M755, M758, M759, M76, M77, M2161, M2162, M65, M66, M67, M700, M7030, M7031, M7032, M71, M7512 |
| Chronic Overlapping Pain Conditions from Schrepf A, Phan V, Clemens JQ, Maixner W, Hanauer D, Williams DA. ICD-10 Codes for the Study of Chronic Overlapping Pain Conditions in Administrative Databases. J Pain. 2020;21(1-2):59-70. | M797, K580, K581, K582, K588, K589, N3010, N3030, N411, N94810, N94818, N94819,  G43001, G43009, G4301, G43011, G43019, G431, G4310, G43101, G43109, G4311, G43111, G43119, G434, G4340, G43401, G43409, G4341, G43411, G43419, G435, G4350, G43501, G43509, G4351, G43511, G43519, G437, G4370, G43701, G43709, G4371, G43711, G43719, G43B, G43B0, G43B1, G43C, G43C0, G43C1, G43D, G43D0, G43D1, G438, G4380, G43801, G43809, G4381, G43811, G43819, G4382, G43821, G43829, G4383, G43831, G43839, G439, G4390, G43901, G43909, G4391, G43911, G43919, G44201, G44209, G44211, G44219, G44221, G44229, M2660, M2662, M2663, S030XXA, M545, M5440, M541, M542, M5489, R5382, S03, S030, S0300, S0300XA, S0300XD, S0300XS, S0301, S0301XA, S0301XD, S0301XS, S0302, S0302XA, S0302XD, S0302XS, S0303, S0303XA, S0303XD, S0303XS, S031, S031XXA, S031XXD, S031XXS, S032, S032XXA, S032XXD, S032XXS, S034, S0340, S0340XA, S0340XD, S0340XS, S0341, S0341XA, S0341XD, S0341XS, S0342, S0342XA, S0342XD, S0342XS, S0343, S0343XA, S0343XD, S0343XS, S038, S038XXA, S038XXD, S038XXS, S039, S039XXA, S039XXD, S039XXS |
| Skin and soft tissue infections separated from Injection Related Infections scheme deployed by Coye AE, Bornstein KJ, Bartholomew TS, et al. Hospital Costs of Injection Drug Use in Florida. Clin Infect Dis. 2021;72(3):499-502. | M726, L03011, L03012, L03019, L03031, L03032, L03111, L03112, L03113, L03114, L03115, L03116, L03119, L03211, L03213, L03221, L03311, L03312, L03313, L03314, L03315, L03317, L03818, L0390, L0201, L0202, L0211, L02211, L02212, L02213, L02214, L02215, L0231, L02411, L02412, L02413, L02414, L02415, L02416, L02419, L02434, L02511, L02512, L02519, L02611, L02612, L02619, L02818, L0291, L0292, L0390, L089, M7989, B372, A480 |
| Blood infections separated from Injection Related Infections scheme deployed by Coye AE, Bornstein KJ, Bartholomew TS, et al. Hospital Costs of Injection Drug Use in Florida. Clin Infect Dis. 2021;72(3):499-502. | A419, R7881, A4101, A4102, A4151, A4152, A4159, A4189, R6521, R6520, A400, A401, A403, A408, A409, A411, A413, A414, A4150 |
| Bone, joint and vascular infections separated from Injection Related Infections scheme deployed by Coye AE, Bornstein KJ, Bartholomew TS, et al. Hospital Costs of Injection Drug Use in Florida. Clin Infect Dis. 2021;72(3):499-502. | I2690, I330, I339, I38, I39, M00011, M00012, M00019, M00021, M00031, M00032, M00051, M00052, M00061, M00062, M00071, M00072, M0009, M00211, M00231, M00232, M00252, M00261, M00272, M00812, M00832, M00851, M00852, M00861, M00862, M00871, M00872, M0088, M0089, M009, M8668, M86071, M8610, M86111, M86112, M86141, M86142, M86151, M86152, M86161, M86162, M86171, M86172, M86179, M8618, M8619, M86272, M86371, M86452, M86452, M86471, M8648, M8649, M8660, M86611, M86612, M86641, M86642, M86651, M86652, M86661, M86662, M86671, M86672, M86679, M8668, M8669, M868X1, M868X4, M868X5, M868X6, M868X7, M868X8, M869 |
| Homelessness | Z590, Z591, Z598 |
| Polysubstance use | F1210, F1211, F12120, F12121, F12129, F12151, F12188, F1220, F12220, F12259, F12288, F12920, F12929, F12950, F12959, F12959, F12988, F1299, F1310, T407X1A, T407X2A, T407X5A, F1520, F1524, F1510, F1594, F1593, F1590, F1514, T43621A, T43622A, T43625A, F16921, F16121, F16159, F1699, F1614, F16229, F1620, F1690, F16129, F1610, F16120, F16251, F1410, F1411, F14120, F14129, F14121, F1414, F14159, F14188, F1419, F1420, F1421, F14220, F14221, F14222, F14229, F1423, F1424, F14288, F1429, F1490, F14920, F14921, F14929, F1494, F1494, F14988, F1499, T405X1A, T405X2A, T405X1A, T405X2A, T405X4A, T405X1S, T405X5A, F1320, F1910, F1290, F19939, F1390, F13239, T424X1A |
| Cocaine use | F1410, F1411, F14120, F14121, F14129, F1414, F14159, F14188, F1419, F1420, F1421, F14220, F14221, F14222, F14229, F1423, F1424, F14288, F1429, F1490, F14920, F14921, F14929, F1494, F1494, F14988, F1499, T405X1A, T405X1A, T405X1S, T405X2A, T405X2A, T405X4A, T405X5A |
| Nicotine dependence | F172, F1720, F17200, F17201, F17203, F17208, F17209, F1721, F17210, F17211, F17213, F17218, F17219, F1722, F17220, F17221, F17223, F17228, F17229, F1729, F17290, F17291, F17293, F17298, F17299, Z720, Z87891 |
| Depression | F33, F330, F331, F332, F333, F334, F3340, F3341, F3342, F338, F339, F3130, F314, F315, F3160, F3164, F319, F322, F323, F3289, F329, F339, F341, F39, F411, F419 |
